# Detecting the Conspecific: Herbivory-Induced Olfactory Cues in the Fall Armyworm (Lepidoptera: Noctuidae)

**DOI:** 10.3390/metabo11090583

**Published:** 2021-08-30

**Authors:** David A. Ingber, Shawn A. Christensen, Hans T. Alborn, Ivan Hiltpold

**Affiliations:** 1 Department of Entomology and Wildlife Ecology, University of Delaware, Newark, DE 19716, USA; 2 United States Department of Agriculture, Agricultural Research Service, Gainesville, FL 32608, USA; shawn.christensen@usda.gov (S.A.C.); hans.alborn@usda.gov (H.T.A.); 3 Agroscope Changins, Entomology in Field Crops and Viticulture, Plant Protection Strategic Research Division, 1276 Nyon, Switzerland

**Keywords:** *Spodoptera frugiperda*, corn, oviposition, HIPV, VOC, metabolites, FAW, management

## Abstract

The fall armyworm (FAW), *Spodoptera frugiperda* (Smith), is a polyphagous pest whose larval feeding threatens several economically important crops worldwide with especially severe damage to corn (*Zea mays* L.). Field-derived resistance to several conventional pesticides and Bt toxins have threatened the efficacy of current management strategies, necessitating the development of alternative pest management methods and technologies. One possible avenue is the use of volatile organic compounds (VOCs) and other secondary metabolites that are produced and sequestered by plants as a response to larval feeding. The effects of conspecific larval feeding on fall armyworm oviposition preferences and larval fitness were examined using two-choice oviposition experiments, larval feeding trials, targeted metabolomics, and VOC analyses. There was a significant preference for oviposition on corn plants that lacked larval feeding damage, and larvae fed tissue from damaged plants exhibited reduced weights and head capsule widths. All larval feeding promoted significantly increased metabolite and VOC concentrations compared to corn plants without any feeding. Metabolite differences were driven primarily by linoleic acid (which is directly toxic to fall armyworm) and tricarboxylic acids. Several VOCs with significantly increased concentrations in damaged corn plants were known oviposition deterrents that warrant further investigation in an integrated pest management context.

## 1. Introduction

The fall armyworm (FAW), *Spodoptera frugiperda* (Smith), (Lepidoptera: Noctuidae) is a highly polyphagous, multivoltine pest of many pasture, turf, and agricultural crops [1,2]. Its larval host range spans over 80 host plants in 23 families [3] including economically important crops such as corn (*Zea mays* L.), cotton (*Gossypium* Spp. L.), sorghum (*Sorghum bicolor* L.), rice (*Oryza sativa* L.), and a variety of pasture and turf grasses [1,4,5]. Larval feeding damage is especially severe in corn, where yield losses can range from 17% to as high as 72% [6,7,8]. The fall armyworm is native to the Americas, where outbreaks have been recorded since the late 1700s [1,2]. However, populations of the pest have spread throughout the Eastern hemisphere over the past several years including Africa, India, Southeast Asia, China, and Australia [9,10,11,12,13,14,15,16].

Fall armyworm populations in corn systems are currently managed worldwide largely through the use of chemical insecticides, as well as transgenic plant hybrids that produce toxins derived from the bacterium *Bacillus thuringiensis* Berliner (Bt) in some locales. The continued efficacy of these management tools is threatened due to many instances of field-derived resistance to several chemicals in the pyrethroid, organophosphate, and carbamate families [17,18,19,20]; and Bt resistance beginning in late 2006 [21].

There are currently very few avenues for commercial fall armyworm management that do not involve the use of conventional insecticides or Bt crop hybrids. However, herbivorous insects are known to respond to herbivore-induced plant volatiles (HIPVs), thus HIVPs that repel oviposition by female fall armyworms may provide a novel avenue for fall armyworm integrated pest management (IPM) and insect resistance management (IRM). For example, Signoretti et al. [22] reported data from a series of Y-tube experiments that female fall armyworms strongly preferred the volatiles of undamaged corn plants over those of plants with larval feeding. The reported preference may be an adaptive behavior to avoid competition between newly hatched and already established larvae [22]. Téllez-Rodriguez et al. [23] later applied this concept in an IRM context by conducting field trials using Bt field-corn and non-Bt refuge plants. It was determined that fall armyworm females exhibited a preference towards oviposition on plants with little insect feeding damage [23]. In this context, the corn plants with less feeding damage were Bt plants, as they generally suffered lower degrees of herbivory due to the activity of their toxins (the presence of Bt does not appear to deter fall armyworm oviposition [24]). This deterrence also extends to mechanical damage [25]. The use of HIPVs to repel female oviposition could be a potential avenue to bolster existing technologies, extending their time of efficacy, or possibly serving as a stand-alone method of pest management.

To better understand why gravid females avoid conspecific damaged plants, we herein measure the effects of non-cannibalistic larval competition in the fall armyworm by examining the effects of conspecific tissue feeding on female oviposition, hatch rates, larval survival, and larval fitness; as well as comparing targeted metabolomic and volatile organic compound (VOC) profiles of corn plants with and without feeding damage. The results herein yield candidate chemicals that may serve as foliar deterrents to fall armyworm feeding and oviposition, bolstering existing technologies or possibly serving as stand-alone management tools.

## 2. Results

### 2.1. Two-Choice Oviposition Experiment 

In examining the effects of conspecific damage on possible oviposition targets it was found that female fall armyworms deposited significantly more egg masses on undamaged control plants than on induced plants with larval feeding (Table 1). The hatch rates of the deposited egg masses did not differ significantly between control and induced corn plants (Table 1).

### 2.2. Feeding Trials 

The feeding trials were conducted to examine the effects of conspecific feeding on fall armyworm larval development. There were no significant differences in the live or dry weights of larvae fed either control or induced corn plant tissue (Table 1). Fall armyworm larvae fed induced corn plant tissue had significantly decreased head capsule widths compared to those fed control plant tissue (Table 1).

### 2.3. Targeted Metabolomic Analyses 

**Figure 1:**
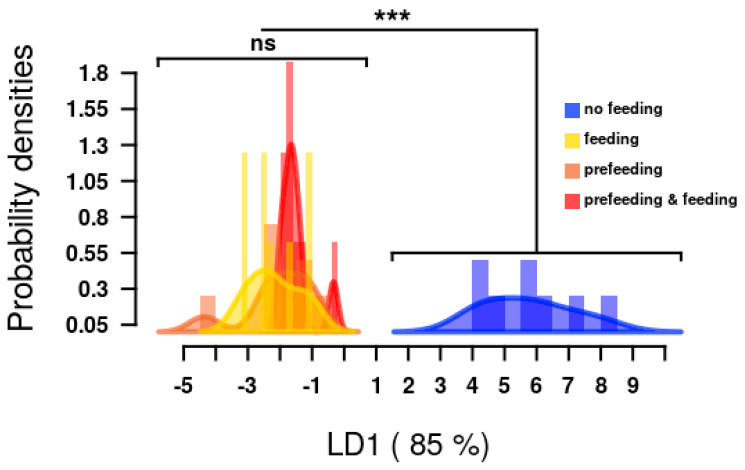
Linear discriminant analysis and change of targeted corn metabolite profiles under four different feeding treatments. Histograms show the distribution of discriminant scores of the metabolomic profiles produced by plants with no feeding (blue), feeding only (yellow), prefeeding only (brown), and prefeeding and feeding together (red). Significant differences between groups of plants are indicated by asterisks, ns: *p* > 0.05 (pairwise Wilcoxon test; *p* < 0.05 after Benjamini and Hochberg correction). The first LD1 explains 85% of the between-group variance.

Analyses were conducted to identify corn plant metabolites that may drive the results of the feeding trials. The first discriminant axis LD1 of the model accounted for 85% of between-group variance (Figure 1). The LD analyses showed a significant difference between the no feeding (control) treatment compared to the remaining three treatments (feeding only, prefeeding only, and prefeeding and feeding together). Larval feeding as a whole affected the targeted metabolomics of maize. Pre-feeding did not result in significant changes to metabolic profiles. (Figure 1)

**Figure 2:**
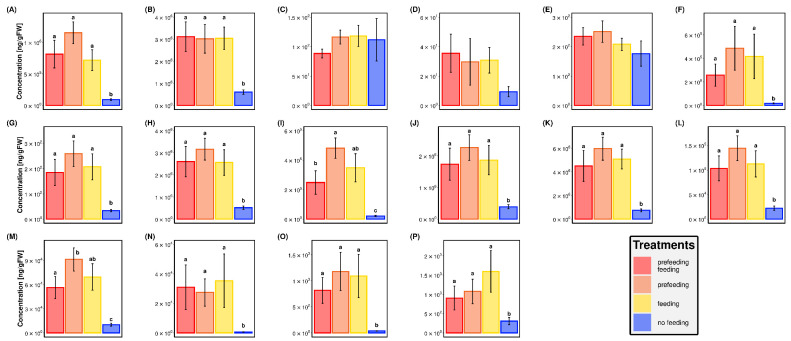
Average concentrations (± Standard Error, *n* = 8) of targeted metabolites. (**A**) linoleic acid, (**B**) tricarboxylic acid, (**C**) salicylic acid, (**D**) trans-jasmonic acid, (**E**) cis-jasmonic acid, (**F**) 6-methoxy-benzoxazolin-2-one, (**G**) indole-3-acetic acid, (**H**) palmitic acid, (**I**) oleic acid, (**J**) steric acid, (**K**) linoleic acid, (**L**) docosanoic acid, (**M**) tetracosanoic acid, (**N**) 12-oxophytodienoic acid, (**O**) cis-10-oxo-11-phytoenoic acid, (**P**) cis-10-oxo-phytodienoic acid. Letters indicate significant differences between treatments. Absence of letters indicates a lack of significance in the overall ANOVA model.

Linoleic acid and tricarboxylic acids (TCAs) were significant discrimination drivers (*p* < 0.0001 and *p* = 0.049, respectively) among the targeted metabolites included in the LD1 model, whereas 6-methoxy-benzoxazolin-2-one, indole-3-acetic acid, palmitic acid, oleic acid, steric acid, linolenic acid, eicosanoic acid, docosanoic acid, tetracosanoic acid, cis-12-oxo-phytodienoic acid, cis-10-oxo-11-phytoenoic acid, and cis-10-oxo-phytodienoic acid each resulted in marginal discrimination (Figure 2).

### 2.4. VOC Analysis

**Figure 3:**
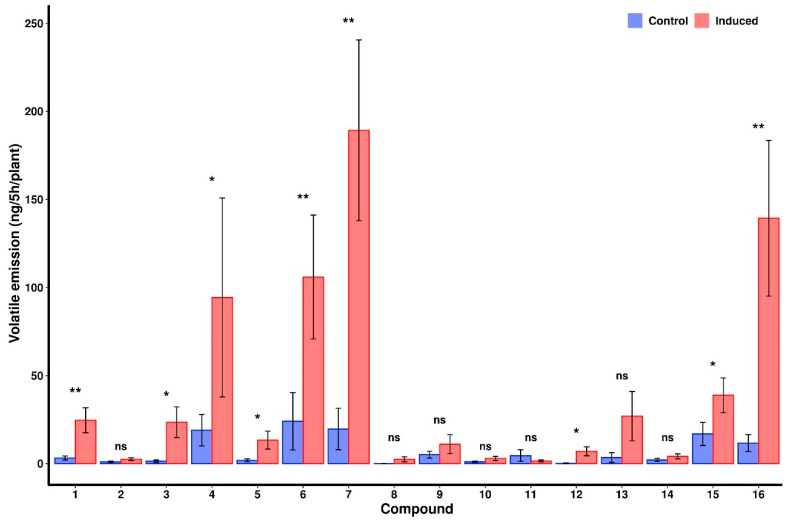
Average nighttime volatile emission rates (+/− S.E., *n* = 11) of 16 compounds from control and induced corn plants, 2 h into the scotophase. Induced plants (red bars) were fed upon by a single second instar fall armyworm larvae for 48 h, control plants received no feeding (blue bars). ns *p* > 0.05, * ≤ 0.05, ** ≤ 0.01. (1) (*E*)-2-Hexanal, (2) β-Myrcene, (3) (*Z*)-3-Hexan-1-ol, (4) (*Z*)-3-Hexenyl Acetate, (5) (*Z*)-β-Ocimene, (6) Linalool, (7) (*E*)-4,8-Dimethyl-1,3,7-Nonatriene, (8) Phenethyl Acetate, (9) Indole, (10) β-Caryophyllene, (11) β-Bergamotene, (12) (*E*)-β-Farnese, (13) α-Humulene, (14) α-Farnesene, (15) (*E*)-Nerolidol, (16) (3*E*,7*E*)-4,8,12-Trimethyl-1,3,7,11-Tridecatetraene.

The VOC profiles of control and induced plants were examined to identify chemical candidates that may be driving the results of the two-choice oviposition experiments. There were no differences in the compounds present in the VOC profiles of control and induced corn plants, though the induced plants produced significantly greater concentrations of volatile organic compounds on average than control plants (*p* < 0.001). There were also significant differences in the concentrations of (*E*)-2-hexanal, (*Z*)-3-hexan-1-ol, (*Z*)-3-hexenyl acetate, (*Z*)-β-ocimene, linalool, (*E*)-4,8-dimethyl-1,3,7-nonatriene (DMNT), (*E*)-β-farnese, (*E*)-nerolidol, and (3*E*,7*E*)-4,8,12-trimethyl-1,3,7,11-tridecatetraene (TMTT) between the two treatment groups (Figure 3).

## 3. Discussion

The significantly reduced egg mass numbers in the two-choice oviposition experiments indicates that conspecific feeding plays a dramatic role in fall armyworm oviposition preferences (Table 1). The published literature on damage-avoiding oviposition in female fall armyworms is limited, with four studies reporting significantly reduced oviposition on damaged corn plants compared with undamaged [22,23,25,26] and one reporting no differences [27]. Larval feeding on induced corn tissue resulted in significantly decreased head capsule widths compared to larvae that were fed control corn tissue (Table 1). The live and dry weight data were both fairly variable, and a larger sample size may have resulted in statistical significance. The availability of larval feeding data is similarly limited; however, Acevedo et al. [28] recently reported that fall armyworm larvae that fed on corn plants with previous feeding damage gained less weight than larvae that fed on undamaged control plants [28]. Despite the dearth of current publications, the majority of the data that are present in published studies indicate that the effects of conspecific feeding in fall armyworm populations is a subject matter that warrants further examination for the identification of alternative management tools for this damaging pest.

Larval feeding in the targeted metabolomics experiment promoted a significant increase in the total concentrations of the targeted metabolites (Figure 1). This is reflected in the data for individual metabolites, which exhibited increased concentrations in response to all larval feeding with the exception of salicylic acid and cis-jasmonic acid, which showed no discernable changes in concentration; and trans-jasmonic acid, which did exhibit increased concentrations compared to control values, but not significantly so (Figure 2). This relative inactivity of these known defensive pathways suggests the possibility that fall armyworm feeding suppresses corn plant production of salicylic and jasmonic acids [29]. It is also interesting to note that the treatment with both prefeeding and feeding combined generally elicited lesser concentrations of the individual metabolites compared to the prefeeding only and feeding only treatments (Figure 2). This difference lacked significance in all of the targeted metabolites except for oleic acid and tetracosanoic acid, which were present in significantly lower concentrations in the combined treatment compared to the prefeeding only treatment but not the feeding only treatment (Figure 2). This generalized response to feeding exhibited by the corn plants does bear the potential to affect the fitness of active feeders, as evidenced by the results of the feeding trials. Linoleic acid has direct toxic effects on fall armyworm larvae [30,31] and it and its derivatives have been reported to cause fitness reductions in other *Spodoptera* [32,33,34]. TCAs consist of a series of organic acids that each possess three carboxylic acid groups and are most notable for their involvement in the citric acid cycle (sometimes referred to as the tricarboxylic acid cycle) of cellular respiration [35]. They also serve as “priming compounds” that induce the production of defensive compounds to combat stressors in plants [36,37].

The effects of corn plant defensive compounds on subsequent, conspecific feeders will be dependent on their persistence in the plant tissues. The lack of significant differences in the metabolomics profiles of the pre-feeding only and post-feeding only treatment groups in the targeted metabolomics analyses indicates that they may persist for at least 48 h. This suggests a lag time between the initial induction of plant defenses due to fall armyworm feeding and the actual establishment of said chemical defenses. Ray et al. [29] determined that fall armyworm frass contains chitinases that suppress plant herbivore defenses and activate pathogen defenses, subsequently increasing larval performance. These chitinases may afford early instar larvae a certain “grace period” to feed and develop to resist plant defenses more effectively. It is also interesting to note that the lack of significant differences in the hatch rates of the egg masses collected in the oviposition trials indicates that these induced plant defenses do not affect the species’ eggs to any significant degree. A follow up study determined that the same chitinases simulated the release increased levels of several HIPV’s including (*E*)-β-farnesene (the aphid alarm pheromone [38]), α-bergamotene, indole, linalool, acetic acid phenyl ester, and oximene, reducing aphid preferences for feeding or chemically induced corn plants when compared to undamaged plants [39].

VOC analyses detected significantly increased concentrations of (*E*)-2-hexanal, (*Z*)-3-hexan-1-ol, (*Z*)-3-hexenyl acetate, (*Z*)-β-ocimene, linalool, (*E*)-4,8-dimethyl-1,3,7-nonatriene (DMNT), (*E*)-β-farnese, (*E*)-nerolidol, and (3*E*,7*E*)-4,8,12-trimethyl-1,3,7,11-tridecatetraene (TMTT) in corn plants induced by larval feeding compared to undamaged control plants (Figure 3). These results are partly consistent with Pinto-Zevallos et al. [40], who conducted similar VOC collections and identified significant differences in the concentrations of thirteen compounds. Pinto-Zevallos et al. [40] and this study both report significant differences in the concentrations (*Z*)-3-hexenyl acetate, (*Z*)-β-ocimene, linalool, DMNT, (*E*)-β-farnese, and TMTT, though there are several differences in the overall VOC profiles and concentrations detected. The differences between the results of this study and Pintos-Zevallos et al. [40] may be due to the use of different corn cultivars, as VOC profiles may vary across corn varieties [41].

The effects of (*Z*)-β-ocimene and Linalool on a variety of insect herbivores are well described, including oviposition deterrent, antifeedant, and parasitoid host location effects, in addition to the triggering of additional defense pathways [42,43,44,45,46,47,48,49,50]. DMNT, (*E*)-β-farnese, (*E*)-nerolidol, and TMTT, have also been reported as exerting oviposition deterrent effects across several insect species, including some Lepidoptera, among other possible effects [51,52,53,54].

(*E*)-2-hexanal, (*Z*)-3-hexan-1-ol, and (*Z*)-3-hexenyl acetate (Figure 3) are “green leaf volatiles” (GLVs), members of a group of compounds comprised of six-carbon alcohols, aldehydes, and esters that are commonly released due to mechanical damage and are perceivable by humans [55,56]. GLVs are often considered as having indirect effects on plant defenses by serving as chemical messengers or priming other plant defenses [57]. However, there is evidence for GLVs serving as direct oviposition deterrents in some insect species [43,58,59]. Therefore, the inclusion of GLVs when considering VOC profiles is still worthwhile.

Pinto-Zevallos et al. [40] also conducted electroantennaegrams on their identified VOCs, in which (*Z*)-3-hexenyl acetate, linalool, DMNT, (*E*)-β-farnese, (*E*)-nerolidol, and TMTT all elicited antennal responses from the fall armyworm. This combined with the compound identification in this study and possible oviposition deterrent effects mark each as prime candidates for examination in IPM and IRM contexts.

A complicating, and often overlooked, aspect of fall armyworm biology in IPM and IRM is the existence of two host strains of the species, the “corn” and “rice” strains [60,61]. The strains are often differentiated from one another through their host preferences [62], though they are not absolute [62,63,64,65]. Several studies have identified differential tolerances in the fall armyworm host strains to several chemical insecticides [18,62,66] and Bt toxins [25,62,67,68,69]. This is upheld by genetic data that depicts significant genetic variation between the corn and rice strains in nuclear and mitochondrial genes affecting detoxification, digestion, and chemoreception [70]. There is a report that larval feeding from the rice strain fall armyworm may induce greater concentrations of defensive compounds than corn strain larval feeding [71]. Therefore, follow up studies should include data on corn and rice strain fall armyworms (as well as corn–rice hybrids [25,67]) to further elucidate plant differential responses to host strain feeding.

## 4. Materials and Methods

### 4.1. Study Insects and Colony Rearing 

Eggs from a corn strain fall armyworm population were obtained from the USDA-ARS station in Gainesville, FL, USA and used to establish a laboratory population. The population originated from Benzon Research Inc. in Carlisle, PA, USA and its corn strain identity was determined by USDA-ARS using the methods of Nagoshi et al. [72]. Four separate colonies of the population were maintained in the laboratory. The development of each colony was staggered by one week to ensure that each life stage was available for use in experiments at any given time. Colonies were all reared following the methods reported in Ingber et al. [67], modified from Perkins [73] and Vélez et al. [74], and all responded equally well to the rearing methods. All life stages were incubated in growth chambers (Model E-54U, Pervical Scientific, Perry, IA, USA) set to 27 °C and a 16:8 L:D photoperiod. Relative humidity was maintained between 50% and 70% by placing large, water-filled containers at the bottom of each chamber. Egg masses were removed from population cages and incubated until larval emergence. Newly emerged neonates were transferred using a soft-tipped paint brush into 32-cell trays (Frontier Agricultural Sciences, Newark, DE, USA) prepared with 5 mL of general-purpose Lepidoptera diet (F9772, Frontier Agricultural Sciences, Newark, DE, USA) in each cell. Trays with larvae were sealed using lids with a thermal adhesive (Frontier Agricultural Sciences, Newark, DE, USA) and incubated until pupation.

Sealed trays were opened to obtain larvae for use in experiments and then resealed. Pupae were removed and placed atop cotton pads (Richmond Dental, Richmond, NC, USA) wet with deionized water in 100 mm petri dishes (Thermo Fisher Scientific Inc., Waltham, MA, USA) that were then placed in 20.3 × 20.3 × 20.3 cm, 6 mm wire mesh hermit crab cages (Florida Marine Research, Sarasota, FL, USA). The cages were fit with covers constructed of 1 mm wire mesh to prevent adult escape, as the 6 mm mesh of the cages themselves was not sufficient to contain smaller moths. A liquid adult diet comprised of flat beer, ascorbic acid, propionic acid, aureomycin, and a vitamin mixture (Vanderzant vitamin mix, Frontier Agricultural Sciences, Newark, DE, USA) [67,73,74] was placed in each cage in a 50 mL Erlenmeyer flask (Thermo Fisher Scientific Inc., Waltham, MA, USA) fitted with a braided cotton wick (Richmond Dental, Richmond, NC, USA) after adult emergence was detected. Sheets of waxed paper (Reynolds Consumer Products, Lake Forest, IL, USA) were placed in between the cage wall and outer mesh cover to serve as an oviposition substrate and were replaced every three days.

### 4.2. Plants

Non-Bt field corn plants (35F38, Source: N3RUS11040-P7, Size: PDF, R3 Batch: 1063498U, Origin: Indiana; Corteva Agriscience, Wilmington, DE, USA) were grown in 15.24 cm diameter containers (TEKU Azalea Style, Poppelmann Plastics USA LLC, Claremont, NC, USA) filled with SunGro Metro-Mix 855 growing mix (SunGro Horticulture, Vancouver, BC, Canada), with one seedling per container, in a greenhouse. This cultivar was selected due to its widespread use in commercial field corn operations. Daily greenhouse conditions ranged between 28 °C and 35 °C and 40% and 70% relative humidity depending on the outside weather conditions. Corn seeds were soaked in a 10% bleach solution for one hour in order to remove any seed treatments. Bleached seeds were rinsed in tap water then left out to dry for at least 24 h prior to planting. Corn plants were grown to approximately the V4–V5 growth stage prior to use in experiments. Cohorts of 20 plants were planted each week to ensure that corn plants of the correct growth stage are always available for use in experiments.

### 4.3. Two-Choice Oviposition Experiment

Fourth instar fall armyworm larvae were placed in the whorl of V6–8 corn plants, one per plant, in the greenhouse. These plants were referred to as “induced” plants, and larvae were allowed to feed for a 48 h period to ensure sufficient feeding damage. Plants with larvae were placed in 60 cm × 60 cm × 75 cm insect rearing tents (Bugdorm, MegaView Science Co. Ltd., Taichung, Taiwan) to prevent larval escape. After the 48 h period elapsed, one induced plant and one undamaged control plant were placed on opposite sides of 200 × 180 × 150 cm pop up mosquito nets (Yoosion Moustiquaire Bed Zipper, ShenZhen Kafan Technology Co. Ltd., ShenZhen, China). A single, gravid, female fall armyworm was released into the mosquito net and left for 96 h to oviposit. Females were removed from rearing cages after 72 h of mass-mating post initial adult emergence. After the oviposition period had elapsed, the plants were removed from the mosquito net and the remaining larval and adult fall armyworm were disposed of. The number of egg masses on each plant was recorded. Egg masses could be of any number of eggs and were considered discrete from one another as long as they were at least 1 cm apart. Twelve cohorts consisting of four mosquito nets each were conducted for a total of 48 repetitions of the two-choice oviposition experiment. Collected egg masses were separated into individual 44.4 mL plastic cups (Solo T125-0090 plastic soufflé portion cups, Solo Cup Company, Hampstead, MD, USA), placed in a growth chamber, and monitored for larval emergence. After larval emergence, the number of hatched eggs and total number of eggs were counted using a Wild M5A stereomicroscope (Wild-Heerbrugg, Heerbrugg, Switzerland) to determine the hatch rates of the deposited egg masses.

### 4.4. Feeding Trials

Induced plants were prepared in the same manner as in the two-choice oviposition experiment. After the 48 h feeding period, the fourth instar larvae were removed from the induced plants. After removal of the original larvae, fresh second instar larvae were placed in the whorls of each induced plant and control plants that received no prior feeding damage. Fall armyworm larvae typically begin feeding on their initial host plant after neonate emergence. Second instar larvae were used to simulate this while simultaneously avoiding the increased mortality rates exhibited by first instars. Plants with larvae were placed in individual 60 cm × 60 cm × 75 cm insect rearing tents (Bugdorm, MegaView Science Co. Ltd., Taichung, Taiwan), where the second instar larvae were left to feed for a 96 h period. The second instar larvae were recovered after the feeding period had elapsed. Twelve cohorts of two induced and two control plants each were conducted for 24 repetitions of each plant type. Thirteen larvae were recovered from induced plants and 14 from control plants. The head capsule widths of recovered larvae were measured using a using a Wild M5A stereomicroscope with a stock 20×, 5:100 measuring eyepiece (Wild Heerbrugg, Heerbrugg, Switzerland). Live weights were measured using an electronic balance (Mettler Toledo AL54, Mettler Toledo LLC, Bristol, PA, USA). After obtaining the live weight measurements, larvae were desiccated using a drying oven (Gravity Oven 180 L, Fisher Scientific Company, LLC, Waltham, MA, USA), then re-weighed to measure their dry weights.

### 4.5. Targeted Metabolomic Analyses

Induced plants were prepared in the same manner as in the previous two experiments, serving as a “prefeeding” phase for this experiment. After the initial induction period had elapsed, fresh second instar larvae were placed into the whorls of a subset of induced and control plants for a second induction period to serve as a “feeding” phase. Therefore, the treatments for this experiment are no feeding, feeding only, prefeeding only, and prefeeding and feeding together. All plants were left undisturbed for a 6 h period after the initiation of the second induction phase after which time 3 cm leaf sections were removed from green, complete leaves from the whorls and outer leaves of each plant and immediately submerged into liquid nitrogen. For quantification of maize metabolites described in this work, samples were solvent extracted, methylated, collected on a polymeric adsorbent using vapor-phase extraction (VPE), and analyzed using GC/isobutane CI-MS as previously described in Schmelz et al. [75]. Metabolite quantification was based on d6-SA (Sigma-Aldrich, St. Louis, MO, USA), d5-JA (C/D/N Isotopes Inc, Pointe-Claire, QC, Canada), or U13C-18:3 (Cambridge Isotope Laboratories Inc., Tewksbury, MA, USA), as internal standards.

### 4.6. Volatile Organic Compound (VOC) Collection and Analysis

V6 maize plants were placed into glass chambers (20 cm diameter, 80 cm height) adapted from Turlings et al. [76]. The pots containing the plants were wrapped into aluminum foil to reduce contamination with the volatile organic compound from the soil/potting mix. The corn plants were induced using the two first instar fall armyworms placed into the whorl. After 24 h, trapping filters (30 mg of HayeSep Q, 4 mm diameter, 8.8 cm long; Volatile Assay Systems, NY, USA) conditioned with 3 mL of dichloromethane (GC-grade; Sigma-Aldrich, MO, USA) were inserted into horizontally connected glass ports and secured with screw cap fittings. The filters were then connected to an air flowmeter and vacuum pump. A Teflon tube (Volatile Assay Systems, Rensselaer, NY, USA) was inserted in a second glass port located at the bottom of the VOC collection chamber (20 cm high, ca. 2 cm above the pot). Purified air entered the system (1.2 L/min) through the Teflon tube and was pulled out of the glass vessel at a rate of 0.6 L/min, through the trapping filters. Odor collection started 2 h into the scotophase and lasted for 3 h. After volatile trapping was completed, the filters were eluted with 200 μL of dichloromethane (GC-grade; Sigma-Aldrich, St Louis, MO, USA) following D’Alessandro et al. [77]. A control consisted of corn plants of the same growth stage, but without induction. A total of 11 samples were collected for each treatment (Induced and Control), and two internal standards were added (*n*-octane and nonyl acetate, 200 ng each in 10 μL dichloromethane; Sigma-Aldrich, St Louis, MO, USA). VOC samples were stored in a freezer at −80 °C prior to their analysis.

Volatiles were analyzed with an Agilent 6890 gas chromatographer (Agilent Technologies Inc., Santa Clara, CA, USA) equipped with a mass spectrometer (Agilent 5973 Network Mass Selective Detector, transfer line 230 °C, source 230 °C, ionization potential 70 eV; Agilent Technologies Inc., Santa Clara, CA, USA). Aliquots of 3 μL of each sample were injected using the pulsed splitless mode into an apolar capillary column (HP-5MS, 30 m, 0.25 mm ID, 0.25 μm film thickness, Agilent Technologies Inc., Santa Clara, CA, USA). Helium was used as the carrier gas at a constant pressure of 1.6 psi, 1.2 mL/min. The column temperature was maintained at 40 °C for 2 min after injection and then gradually increased to 100 °C at a rate of 9 °C/min, then again to 200 °C at 6 °C/min. Volatiles were putatively identified by comparison of their mass spectra with those of the NIST 08 library. The relative concentrations of the compounds were estimated by comparison to those of the internal standards.

### 4.7. Data Analyses

Data comparing the control and induced groups in both the two-choice oviposition experiments and feeding trials were compared in SAS 9.4 (SAS Institute Inc., Cary, NC, USA). Data pertaining to the targeted metabolomic and HIPVs profiles of control and induced plants were compared in R 4.0.5.

The mean number of egg masses deposited on control and induced plants were compared using a generalized linear model with a Quasi-Poisson distribution (PROC GLIMMEX) as the number of egg masses deposited on each plant within each trial were not independent from one another [78]. The mean proportion of hatched eggs from masses collected from control and induced plants were compared with a *t*-test (PROC TTEST). T-tests were also employed to compare the mean live and dry weights, and head capsule widths of surviving larvae in the feeding trials.

A linear discriminant analysis (LDA) was conducted to determine the effects of the four treatments (no feeding, feeding only, prefeeding only, and feeding and prefeeding together) on the targeted corn plant metabolites. The assumption of homogeneity was met in permutations (permutation test following Kergunteuil et al. [79], permutation = 999, F = 0.0.99, *p* = 0.431). The differences in the distribution of the metabolic profiles along LD1 were tested using a pairwise Wilcoxon signed-rank test with a Benjamini–Hochberg adjustment to the level of significance. The concentrations of individual metabolites between treatment groups were compared using one-way ANOVA followed by Tukey–Kramer adjusted pairwise comparisons if the overall model was significant.

Data comparing the collected volatile profiles of induced and control plants as well as the total average VOC concentrations of each plant type were analyzed using two-sample *t*-tests.

## 5. Conclusions

The present study partially elucidates why FAW gravid females avoid plants damaged by conspecifics due to the plants emitting possibly repellent VOCs and affecting the development of their progeny. The outcomes of this research may offer several HIPVs that could be used as foliar deterrents and eventually be incorporated into new transgenic plant varieties [80]. Further inquiries could include host strain specific data, foliar treatments using identified metabolites, additional feeding trial time points (e.g., 24 h and 72 h), and leaf damage values to correlate with larval growth factors. The timely production of novel management techniques could be a large boon for their agricultural practices and industries by providing a safe and effective means of pest management.

## Figures and Tables

**Table 1:** Oviposition, hatch, and larval growth data from two-choice oviposition experiments and feeding trials.

Parameter	Control ^a^	Induced ^a^	df ^b^	*t*-Value	*p*-Value
Egg Masses ^c^	0.47 ± 0.15	0.09 ± 0.06	92	2.17	0.0325
Hatch Rate	0.87 ± 0.03	0.93 ± 0.01	24	−0.98	0.3373
Live Weight ^d^	8.01 ± 1.17	4.68 ± 1.09	25	1.61	0.1201
Dry Weight ^d^	1.01 ± 0.14	0.67 ± 0.15	25	1.65	0.1112
HCW ^e,f^	1.00 ± 0.06	0.77 ± 0.05	25	3.02	0.0057

^a^ Mean ± Standard Error. ^b^ Degrees of freedom representing each individual egg mass across all replicates. ^c^ Generalized Linear Model with Quasi-Poisson distribution. ^d^ Measured in mg. ^e^ Head Capsule Width. ^f^ Measured in mm.

## Data Availability

Data supporting the reported results are in the possession of D.A.I. and I.H. and are available upon request. Data are also publically available through MDPI.
